# Bio-Mapping of Microbial Indicators to Establish Statistical Process Control Parameters in a Commercial Beef Processing Facility

**DOI:** 10.3390/foods11081133

**Published:** 2022-04-14

**Authors:** David A. Vargas, Karla M. Rodríguez, Gabriela K. Betancourt-Barszcz, Manoella I. Ajcet-Reyes, Onay B. Dogan, Emile Randazzo, Marcos X. Sánchez-Plata, Mindy M. Brashears, Markus F. Miller

**Affiliations:** 1 International Center for Food Industry Excellence, Department of Animal and Food Sciences, Texas Tech University, Lubbock, TX 79409, USA; andres.vargas@ttu.edu (D.A.V.); karla-melissa.rodriguez@ttu.edu (K.M.R.); gabbetan@ttu.edu (G.K.B.-B.); majectre@ttu.edu (M.I.A.-R.); onay.dogan@ttu.edu (O.B.D.); mindy.brashears@ttu.edu (M.M.B.); markus.miller@ttu.edu (M.F.M.); 2 Nebraska Beef Ltd., Omaha, NE 68107, USA; edazzo@nbeef.com

**Keywords:** microbial SPC, beef bio-mapping, microbial interventions, indicator microorganisms

## Abstract

The objective was to conduct a bio-mapping of microbial indicators to determine statistical process control (SPC) parameters at a beef processing plant to establish microbiological baselines and process control parameters to support food safety management decisions. EZ-Reach^TM^ swabs were used to collect 100 cm^2^ area samples at seven different locations throughout the beef processing line at four different regions on the carcass. Each of the eight sampling days evaluated included three samples collected per sampling location/carcass region for a total of 84 samples per day. Enumeration of total aerobic bacteria, Enterobacteriaceae, and *Escherichia coli* was performed on each sample. Microbial SPC parameters were estimated for each sampling point. Statistical differences between sampling points for all carcass locations *(p <* 0.001) followed an overall trend with higher values at pre- and post-evisceration with a continuous decrease until final interventions with a slight increase in counts during the chilling process and a final increase after fabrication. Variability at sampling points is the result of the nature of the process and highlights open opportunities for improvement of the food safety system. Microbial baselines and SPC parameters will help support decision making for continuous process improvement, validation of intervention schemes, and corrective action implementation for food safety management.

## 1. Introduction

Ever since the *Escherichia coli* O157:H7 outbreak in United States ground beef during 1992–1993, the meat industry and regulatory agencies changed completely the way of processing and inspection of red meat. The U.S. industry and government focused their attention on reducing the risk of pathogen presence on carcasses and products that can cause foodborne illness due to the consumption of red meats. According to the Interagency Food Safety Analytics Collaboration (IFSAC), in 2012 the attribution of foodborne illnesses to *Escherichia coli* O157:H7 from beef was close to 46%, being the highest contributor for that year [1]. The same report released for 2019 indicates an attribution of about 23%, being the second highest contributor after Vegetable Raw Crops [2], demonstrating that there is a clear decline in the number of cases that can be linked to beef meat consumption. This progress by the beef industry is the result of multiple initiatives, including the evaluation and validation of a series of antimicrobial interventions schemes to reduce the presence of certain pathogens, the improvement of sanitary dressing procedures during the slaughter process, the voluntary guidance documents, and requirements implemented by the regulatory agencies, and the improvement in processing technologies not only for the slaughter process but also for microbial sampling, detection, and quantification [3,4,5,6,7].

Multiple aspects have changed in the regulatory requirements established by the U.S. Department of Agriculture’s Food Safety and Inspection Service (USDA-FSIS) to control the presence of *Escherichia coli* O157:H7 [8]. The zero tolerance regulation for *Escherichia coli* O157:H7 in ground beef and trim, the mandated implementation of the Hazard Analysis and Critical Control Point (HACCP) systems, the addition of six non-O157 Shiga-Toxin producing *Escherichia coli* serogroups to the list of adulterants in beef, and the discussion about potential new performance standards for *Salmonella* spp. are some of the key events that incentivize the U.S beef industry to continuously evaluate and implement the use of antimicrobial interventions during the beef harvest [3]. Antimicrobial interventions have been found to be very effective in the reduction of pathogens in meat and nowadays a series of sequential physical and chemical interventions are commonly used by the beef industry as one of the main mechanisms to control the presence of harmful bacteria along with proper sanitary dressing procedures [4,9,10,11,12].

Live cattle are carriers of pathogenic microorganisms which later can contaminate carcasses during slaughter in commercial processing settings. Moreover, beef is an animal product that features adequate conditions like pH, water activity and nutrient content to not only become contaminated but also to support the growth of these microorganisms during storage and handling that may result in important public health threats. Researchers have indicated that there are three key issues in the production of meat products that have an importance on the risk of contamination: (1) pathogen levels on hides, (2) correct removal of hides minimizing cross-contamination between hides and carcass, and (3) efficacy of antimicrobial interventions during the slaughtering process [3].

Multiple factors can affect indicator and pathogen levels on hides such as the season, where higher levels of pathogens can be found during warmer months, or the production system, where animals on feedlots, due to closeness between the animals, present higher fecal contamination on hides when compared with animals on grazing systems [13]. Despite these differences, hide contamination will always be a problem as it is an intrinsic part of commercial production systems. There is promise in the implementation of pre-harvest interventions that could possibly reduce the incoming loads of pathogens arriving at the processing facility, as well as interventions before slaughtering, such as hides washes that can contribute to microbial reductions [14]. Furthermore, the correct removal of hides avoiding cross-contamination between hides and carcasses by the implementation of sanitary dressing procedures is critical as proper training and application of best practices significantly reduce the levels of pathogens and microbial indicators on carcasses [15]. Recurrent employee training, supervision, and practical demonstrations of how bacterial transfer occurs and the importance of good hygiene practices in combination with microbial testing for process validation are some of the strategies followed by the industry to reduce the risk of cross-contamination and comply with regulations.

The efficacy of antimicrobial interventions during the slaughtering process is one of the key drivers for pathogen reduction during production of meat in the United States. Consumers expect that foods in general are free of foodborne pathogens and have a reasonable shelf-life, where interventions play a huge role [16]. According to the Food and Drug Administration, antimicrobials are chemicals that, when added to food, prevent or retard deterioration [17]. Application of antimicrobials is never a substitute for implementing good sanitation practices in slaughtering plants, since adequate practices have been proven to reduce initial counts of indicators and pathogens [11]. Numerous antimicrobial interventions have been described in the literature to be effective against foodborne pathogens found in red meats and investigations to find new antimicrobials or improve the use of the ones that are already in practice are still topics of interest for the beef industry [4,9,12,18,19].

Bio-mappings of microbial indicators and determinations of statistical process control parameters are proven tools that beef processing operations have available to evaluate the performance of their sanitary practices and interventions, and to investigate if their process is under control [20,21]. By control, it is understood that the number and the level of specific interventions are actually reducing microbial loads in final product to a level where it is safe and contributes to providing a reasonable shelf-life despite of the variation on microbial loads that may exist in the process. The basic principle of control charts is related with the intrinsic characteristic that any process has variation, and the common causes of this variation are difficult to specify [22]. When these common causes are the only causes of variation, the process is established as in statistical control. Special factors can also contribute to this variability, in microbial control charts, machines malfunctioning, variation in antimicrobial concentrations, operator errors, and differences in shifts are among the most common of these factors [22]. When these special causes are the cause of variation, the process is to be said as out of statistical control. There are several types of charts for continuous variables such as mean, standard deviation, and range charts as the most common ones. The implementation of this tool can lead to a continuous monitoring process with identification of possible problems before it actually happens.

The goal of this study was to compare microbial data collected periodically with historical data and determine if the process variation is in control (consistent) or if it is out of control (unpredictable) [23]. Because of the variability of microbial populations and potential pathogen levels that exists in animals that enter to beef processing plants, the objective of this study was to establish a practical bio-mapping protocol to quantify the microbial levels throughout the whole process in a commercial beef processing facility and perform statistical analysis to establish process control parameters that can be used for food safety management in the plant. In addition, bio-maps can be used to establish microbial baselines for comparison with processing changes in processing facilities, such as new antimicrobial interventions, changes in the process steps, or modification of processing speed lines.

## 2. Materials and Methods

### 2.1. Beef Processing Plant Interventions

Swabs collected for this study were obtained from a large commercial USDA-FSIS inspected beef processing facility located in the U.S. This plant operates in two shifts and processes around 1800 heads of cattle per day. During beef slaughter, the microbial interventions applied consist of a steam vacuum after de-hiding at the inside round area, hock, and shank with steam at 82 ± 5 °C, application of a carcass water wash at room temperature before evisceration (pre-evisceration), followed by a 2–5% concentration lactic acid spray treatment at 49 ± 5 °C applied in a uniform spray pattern for coverage of the whole carcass at a pressure >15 psi. After evisceration and splitting (post-evisceration), a carcass water wash at room temperature was applied, followed by a lactic acid spray treatment at 2–5% concentration at 49 ± 5 °C, applied in a cabinet at a pressure >15 psi. Subsequently, trimming and visual inspection along the line is performed (final rail) until reaching a hot water whole carcass wash at 85 ± 5 °C plus a lactic acid spray treatment (2–5%) at 49 ± 5 °C applied in a uniform spray pattern in the whole carcass at a pressure >15 psi (after interventions). After final interventions, carcasses are held in a cooler for 18–24 h, reaching a surface temperature at the end of the chilling period of <7 °C (after chilling). Then, carcasses go through a lactic acid spray cabinet (2–5%) with an operating pressure >15 psi and temperature around 49 ± 5 °C while exiting the holding cooler and prior to the entry into the fabrication room (cold carcass). Finally, a lactic acid spray (2–5%) with an operating pressure >15 psi and temperature around 49 ± 5 °C is applied on each of the different sub-primals processing lines (after fabrication). A total of 7 locations in the plant were evaluated.

### 2.2. Sample Collection

EZ-Reach™ 25 mL buffered peptone water (BPW) swabs (World Bioproducts, Mundelein, IL, USA) were collected using a 3M^TM^ cattle 100 cm^2^ template (3M, Saint Paul, MN, USA) at each of the seven different sampling locations in the beef processing line (pre-evisceration, post-evisceration, final rail, after interventions, after chilling, cold carcass, and after fabrication) on four carcass regions (shank, inside round, midline, and brisket). Each sampling day included three samples per sampling location/carcass region, for 7 sampling locations and 4 carcass regions, resulting in a total of 84 samples per day. Samples were collected during eight different days of processing, equally spaced in two months, to account for lot variations, intrinsic process variability, and statistical process control analysis during summer 2021. Sampling locations were determined due to the highest likelihood of contamination or the presence of an intervention step in the process.

### 2.3. Swab Processing

Swabs were immediately chilled and shipped overnight to the ICFIE Food Microbiology Laboratory at Texas Tech University for microbiological analysis. Upon arrival, swabs were homogenized in a stomacher (Model 400 circulator, Seward, West Sussex, UK) at 230 rpm for one minute. Homogenates were serially diluted in 9 mL BPW (Millipore Sigma, Danvers, MA, USA) tubes and plated for aerobic plate counts (APC), Enterobacteriaceae (EB), and *Escherichia coli* (EC). Samples were enumerated using the TEMPO^®^ system (BioMérieux, Paris, France) following the AOAC 121.204 official method for enumeration of aerobic plate counts, AOAC 050.801 for Enterobacteriaceae, and AOAC 080.603 for *Escherichia coli*. All cards were incubated at 35 ± 1 °C for 24 ± 2 h.

### 2.4. Statistical Analysis

All data were analyzed using R (Version 4.1.2) statistical software to evaluate the change in microbial loads at different process locations (pre-evisceration, post-evisceration, final rail, after interventions, after chilling, cold carcass, and after fabrication) throughout the beef processing line for each of the four carcass regions (shank, inside round, midline, and brisket). Counts were transformed into Log CFU/cm^2^ for APC and Log CFU/sample for EB and EC to facilitate data visualization. A one-way ANOVA analysis was performed, comparing counts at each of the different steps in the beef processing line for each carcass region, followed by pairwise comparison T-test adjusted by the Benjamin and Hochber method. If parametric assumptions were not met, the Kruskal–Wallis test was used as a non-parametric alternative for the ANOVA, followed by a pairwise multiple comparison Wilcoxon’s test adjusted by the Benjamin and Hochber method. All significant differences were evaluated using a *p*-value lower than 0.05.

Furthermore, the Shewart’s expected average statistical quality control chart ($\bar{X}$ chart) was developed to estimate statistical process control parameters for each of the different steps during the beef processing line. The parameters were estimated based on a per carcass basis; thus, swabs from different carcass regions from the same carcass were used as different samples taken per day. The center line or grand average ($\overset{=}{X}$) parameter was estimated by calculating an average per day (n = 12 samples) and then, the average of each of the eight days means was estimated for each of the different sampling points locations in the beef processing line. The other two parameters, upper control limit (UCL) and lower control limit (LCL) for each sampling point were estimated by the use of equation 1 and 3 where the average standard deviation ($\bar{S}$) was used and an A_3_ factor (0.866), given that 12 samples were collected on each sampling day during 8 eight days [22]. In addition, the average run length (ARL) parameter was estimated using equation 4 by dividing one to the probability (p) that any given point will be out of the control limits for each sampling point.


(1) $$\mathrm{Upper}\;\mathrm{Control}\;\mathrm{limit}\;(\mathrm{UCL})=\overset{=}{X}+A_{3}*\bar{S}$$

(2) $$\mathrm{Central}\;\mathrm{Line}\;(\mathrm{Grand}\;\mathrm{Average})=\overset{=}{X}\;\;\;\;\;$$

(3) $$\mathrm{Upper}\;\mathrm{Control}\;\mathrm{limit}\;(\mathrm{UCL})=\overset{=}{X}-A_{3}*\bar{S}$$

(4) Average Run Length (ARL)=1/p   

## 3. Results

Aerobic plate counts were reported in Log CFU/cm^2^ basis, while Enterobacteriaceae and *Escherichia coli* counts were transformed to Log CFU/sample for statistical analysis. For Enterobacteriaceae and *Escherichia coli* counts, some of the counts were below 1 CFU/cm^2^, resulting in negative values when transformed to Log CFU/cm^2^, thus making visualization hard to interpret. Log CFU/sample counts were achieved by multiplying the CFU/cm^2^ counts by 100 cm^2^ of area sampled and then log_10_ converted, resulting in Log CFU/100cm^2^ measurements, which are equivalent to Log CFU/sample.

### 3.1. Aerobic Plate Counts (APC)

**Figure 1:**
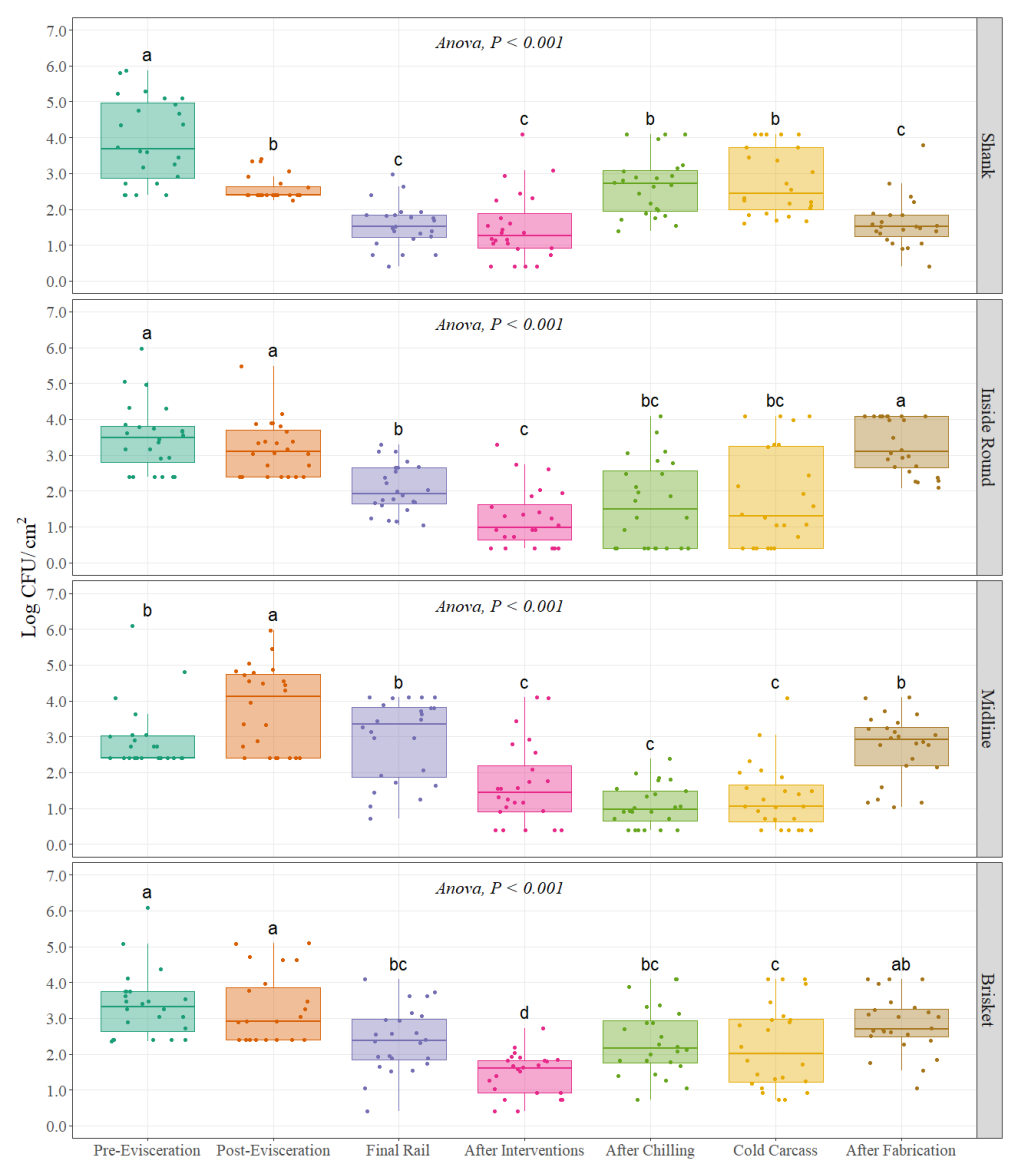
Mesophilic aerobic plate counts (Log CFU/cm^2^) at different carcass regions throughout different sampling locations of the beef processing line (n = 24 swabs per carcass region/sampling location). In each boxplot, the horizontal line crossing the box represents the median, the bottom and top of the box are the lower and upper quartiles, the vertical top line represents 1.5 times the interquartile range, and the vertical bottom line represents 1.5 times the lower interquartile range. ^(a–d)^ For each individual carcass region, boxes with different letters are significantly different according to ANOVA analysis followed by a pairwise comparison *t*-test at *p* < 0.05. The points represent the actual data points.

For aerobic plate counts, interventions and dressing practices throughout the beef processing line significantly change the microbial loads at each of the four different carcass regions (*p* < 0.001), (Figure 1). An overall trend for aerobic plate counts was observed with higher values at pre- and post-evisceration with a continuous decrease until final interventions on the carcass (After Interventions) with a slight increase in counts during the chilling process (Cold Carcass) and a final increase after fabrication. Initial counts at the pre-evisceration step were 3.93, 3.49, 2.94, and 3.38 Log CFU/cm^2^ at shank, inside round, midline, and brisket, respectively (Table 1). Aerobic plate counts at the midline region in the carcass at pre-evisceration were significantly lower than the other three carcass regions (p = 0.011), being the highest at shank followed by inside round, and brisket. The lowest counts found during the entire process were after final interventions (After Interventions) with 1.49, 1.24, 1.44 Log CFU/cm^2^ for shank, inside round, and brisket, respectively, while for midline it was after chilling with 1.09 Log CFU/cm^2^ (Table 1). Counts at the four carcass regions were significantly different *(p <* 0.001) at the end of the slaughtering process (Cold Carcass) when compared with pre-evisceration, with reductions of 1.16, 1.68, 1.64, and 1.15 Log CFU/cm^2^ for shank, inside round, midline, and brisket, respectively, (Figure 1), (Table 1). Aerobic plate counts were significantly higher after fabrication when compared with the previous step for inside round, midline, and brisket with an increase of 1.45, 1.42, 0.59 Log CFU/cm^2^, while for shank counts were reduced by 1.17 Log CFU/cm^2^ (Figure 1), (Table 1). 

### 3.2. Enterobacteriaceae Counts (EB)

**Figure 2:**
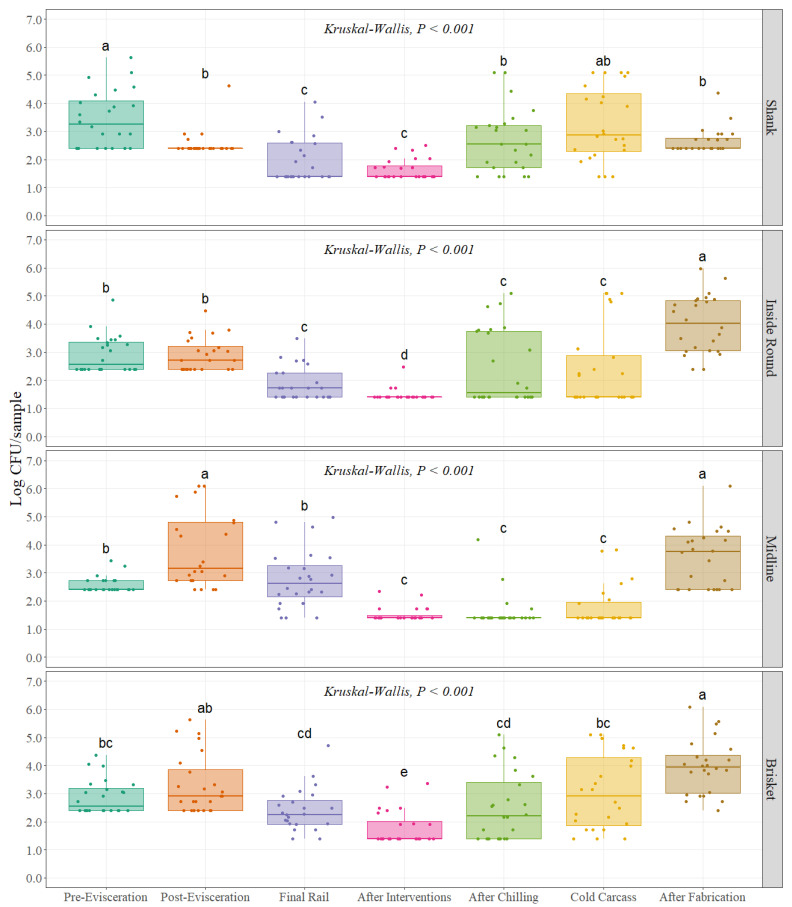
Enterobacteriaceae counts (Log CFU/sample; equivalent to Log CFU/100 cm^2^) at different carcass regions throughout different sampling locations of the beef processing line (n = 24 swabs per carcass region /sampling location). In each boxplot, the horizontal line crossing the box represents the median, the bottom and top of the box are the lower and upper quartiles, the vertical top line represents 1.5 times the interquartile range, and the vertical bottom line represents 1.5 times the lower interquartile range. ^(a–d)^ For each individual carcass region, boxes with different letters are significantly different according to Kruskal–Wallis analysis followed by a pairwise comparison Wilcoxon’s test at *p* < 0.05. The points represent the actual data points.

For Enterobacteriaceae counts, a non-parametric approach test was used for analysis, as this type of test do not need any assumption about specific distributions of the data. The distribution of the data, especially after interventions and after chilling, suggests a non-normal distribution due to consistently lower counts related with limit of quantification (LOQ = 25 CFU/sample). When assumptions are not met for performing parametrical analysis, a Kruskal–Wallis test was preferred instead of ANOVA test to find differences between sampling points throughout the beef processing line. Interventions and dressing practices throughout the beef processing line significantly changed the Enterobacteriaceae counts at each of the four different carcass regions *(p <* 0.001), (Figure 2). A similar trend observed for APC was also observed for Enterobacteriaceae counts with higher values at pre- and post-evisceration with a continuous decrease until final interventions on the carcass (After Interventions) with a slight increase in counts during the chilling process (Cold Carcass) and a final increase after fabrication. Initial counts at the pre-evisceration step were 3.47, 2.93, 2.57, and 2.88 Log CFU/sample at shank, inside round, midline, and brisket, respectively (Table 1). Enterobacteriaceae counts at the midline region of the carcass at pre-evisceration were significantly lower than the other three regions *(p =* 0.002), being the highest at shank and followed by inside round and brisket. The lowest counts found during the entire process were after final interventions (After Interventions) with 1.65, 1.47, 1.53, and 1.79 Log CFU/sample for shank, inside round, midline, and brisket, (Table 1). Counts at two out of the four carcass regions were significantly different *(p <* 0.001) at the end of the slaughtering process (Cold Carcass) when compared with pre-evisceration, with reductions of 0.51 and 0.78 Log CFU/sample for inside round and midline, while no difference was found for shank and brisket (Figure 2), (Table 1). Enterobacteriaceae counts were significantly higher after fabrication when compared with the previous step for inside round, midline, and brisket with an increase of 1.58, 1.76, 0.90 Log CFU/sample, while for shank, counts were not statistically different (Figure 2), (Table 1).

### 3.3. Escherichia coli Counts (EC)

**Figure 3:**
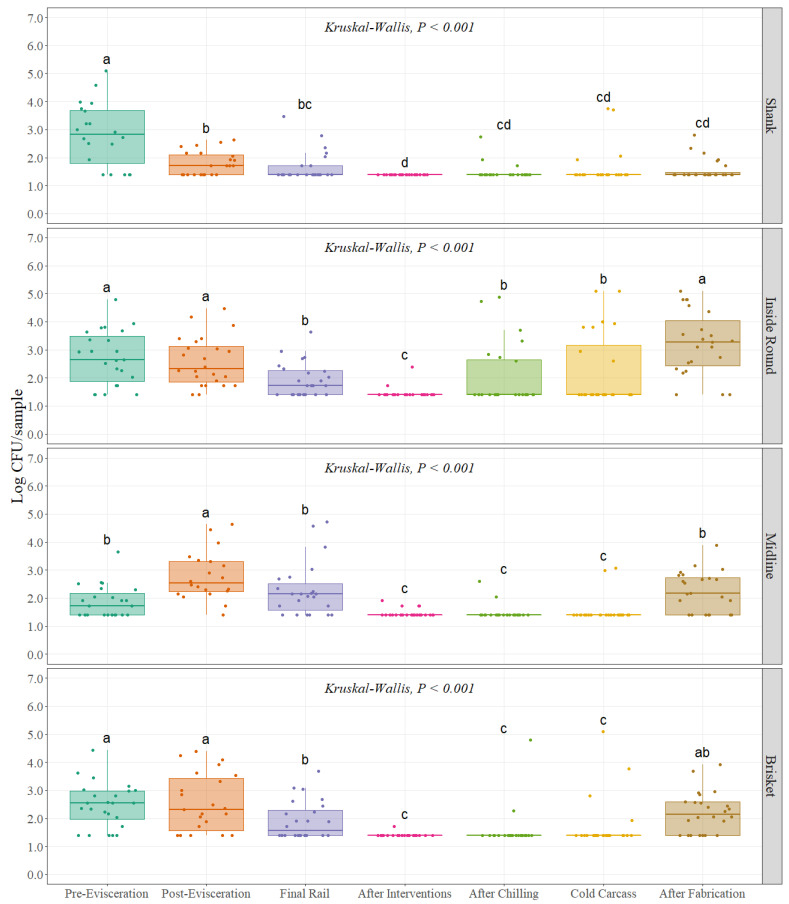
*Escherichia coli* counts (Log CFU/sample; equivalent to Log CFU/100 cm^2^) at different carcass regions throughout different sampling locations of the beef processing line (n = 24 swabs per carcass region/sampling location). In each boxplot, the horizontal line crossing the box represents the median, the bottom and top of the box are the lower and upper quartiles, the vertical top line represents 1.5 times the interquartile range, and the vertical bottom line represents 1.5 times the lower interquartile range. ^(a–d)^ For each individual carcass region, boxes with different letters are significantly different according to Kruskal–Wallis analysis followed by a pairwise comparison Wilcoxon’s test at *p* < 0.05. The points represent the actual data points.

For *Escherichia coli* counts, same approach as Enterobacteriaceae counts was followed due to specific distribution of the data. Interventions and dressing practices throughout the beef processing line significantly change EC counts at each of the four different carcass regions *(p <* 0.001), (Figure 3). A similar trend found in EB was obtained for EC with higher values at pre- and post-evisceration with a continuous decrease until final interventions on the carcass (After Interventions) with a slight increase in counts during the chilling process (Cold Carcass) and a final increase after fabrication. Initial counts at the pre-evisceration step were 2.83, 2.72, 1.87, and 2.47 Log CFU/sample at shank, inside round, midline, and brisket, respectively (Table 1). *Escherichia coli* counts at the midline region of the carcass at pre-evisceration were significantly lower than shank and inside round regions *(p =* 0.001), being the highest at shank and followed by inside round, and brisket. The lowest counts found during the entire process were after final interventions (After Interventions) with 1.40, 1.45, 1.46, and 1.41 Log CFU/sample for shank, inside round, midline. and brisket, (Table 1). Counts at the four carcass regions were significantly different *(p <* 0.001) at the end of the slaughtering process (Cold Carcass) when compared with pre-evisceration, with reductions of 1.18, 0.49, 0.34, 0.74 Log CFU/sample for shank, inside round midline, and brisket (Figure 3), (Table 1). Enterobacteriaceae counts were significantly higher after fabrication when compared with the previous step for inside round, midline, and brisket with an increase of 0.99, 0.69, 0.49 Log CFU/sample, while for shank counts were not statistically different (Figure 3), (Table 1).

### 3.4. Statistical Process Control

**Figure 4:**
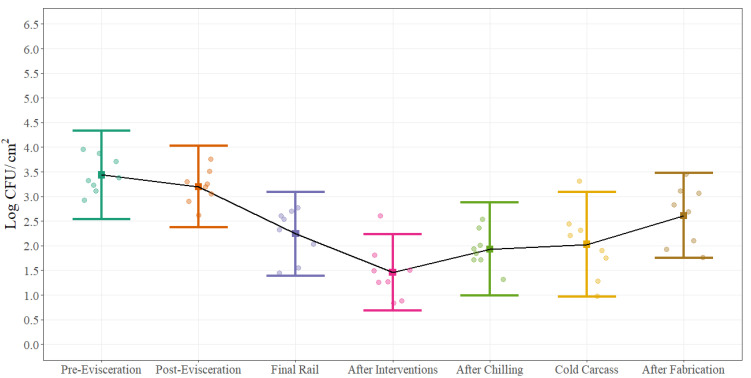
Statistical microbial process control for Aerobic Plate Counts (Log CFU/cm^2^) at different sampling locations on the beef processing line. Solid squares represent the mean of each sampling point for all carcass regions and error bars represent ±3 standard deviations around the mean using a sample size of 12 swabs collected during 8 different sampling days. The black solid line represents the change on the average of each sampling point throughout the beef processing line. The points represent the actual data points.

**Figure 5:**
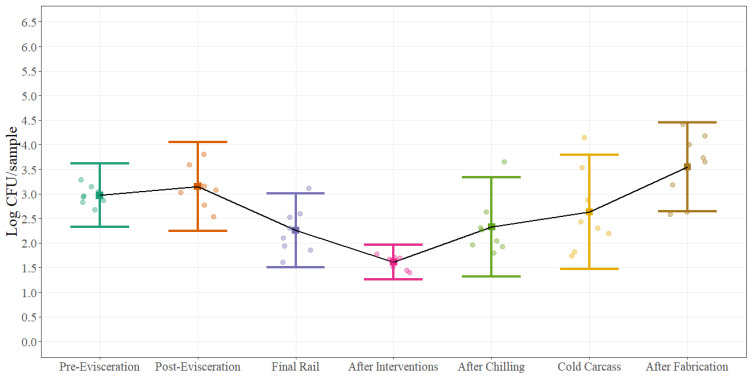
Statistical microbial process control for Enterobacteriaceae (EB) (Log CFU/sample; equivalent to Log CFU/100 cm^2^) at different sampling locations on the beef processing line. Solid squares represent the mean of each sampling point for all carcass regions and error bars represent ±3 standard deviations around the mean using a sample size of 12 swabs for each microorganism. The black solid line represents the change on the average of each sampling point throughout the beef processing line for each microorganism. The points represent the actual data points.

**Figure 6:**
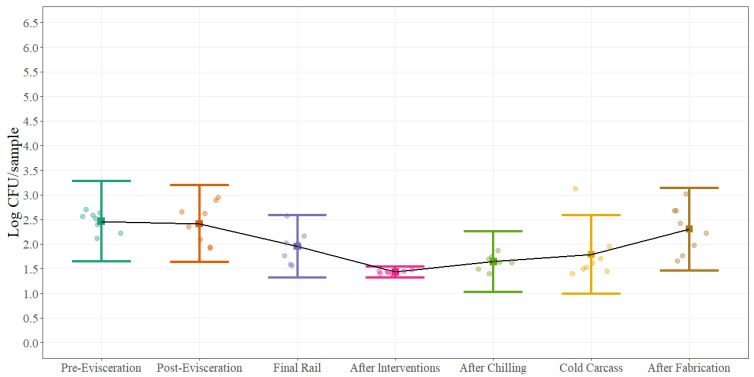
Statistical microbial process control for *Escherichia coli* (EC) (Log CFU/sample; equivalent to Log CFU/100 cm^2^) at different sampling locations on the beef processing line. Solid squares represent the mean of each sampling point for all carcass regions and error bars represent ±3 standard deviations around the mean using a sample size of 12 swabs for each microorganism. The black solid line represents the change on the average of each sampling point throughout the beef processing line for each microorganism.

Statistical process control parameters were estimated for aerobic plate counts, Enterobacteriaceae and *Escherichia coli* counts. Process control parameters are presented as a chart and table for aerobic plate counts (Figure 4), (Table 2) at each of the different sampling locations while for Enterobacteriaceae (Figure 5) and *Escherichia coli* (Figure 6) only process control charts are presented. The microbial process control graphs were constructed by using the mean of each sampling point represented by the solid square and error bars calculated with ±3 standard deviations (UCL and LCL) around the mean using a sample size of 12 swabs collected during eight different sampling days. It is expected that around 99% of the times, the average for each of the sampling points will fall inside the error bars when the process is under control. The black solid line represents the change on the average of each sampling point throughout the beef processing line. The summary table was constructed by using the mean of each of the sampling points and ±1, ±2, and ±3 standard deviations around the mean. The width of the space between the horizontal lines demonstrates the natural variation that exists in each location in a commercial process. For aerobic plate counts (Figure 4), the variation is similar throughout the beef processing line with very few counts outside the established control parameters.

In contrast, looking at the microbial process control charts for Enterobacteriaceae (Figure 5) and *Escherichia coli* (Figure 6) the natural variation between the different steps throughout the beef processing line is different. As an example, the range between the UCL and LCL at post-evisceration for EB is around 1.80 Log CFU/sample while at After Intervention is 0.71 Log CFU/sample. The natural variation between steps can be highly associated with the process itself. At post-evisceration, the entire gastrointestinal tract is removed from the animal and depending on how well this step is performed EB counts can change dramatically due to re-contamination, while after interventions the narrower range explains the importance of this antimicrobial application step at keeping EB counts low in the process. The same natural variation can be looked at *Escherichia coli* counts (Figure 6) but with an even more dramatic difference between post-evisceration and after interventions explained by the effectiveness of the antimicrobial used on this specific microorganism and the limits of quantification of the technology used for enumeration. Furthermore, wider ranges are seen at final steps of the process because of the series of processing activities that need to be followed before actual completion. Minimum differences such as initial microbial load, inconsistent sanitary dressing procedures, cross contamination, swabbing technique, swabbing area, and antimicrobial concentration can have an impact on the natural variation of final steps leading to bigger gaps between the UCL and LCL and multiple outliers.

Statistical process control charts can provide useful information about how a process is managed and how processors can target corrective actions strategically in order to improve the overall process. As an example, counts after interventions for all three-indicator microorganisms are the lowest for most of the four different carcass regions; however, after the chilling process and after fabrication, counts increase to a level that reach same microbial loads as pre- and post-evisceration. This increase can be explained by the potential recovery of cells after 18–24 h of chilling that were injured after the chemical intervention applications that were not recovered during the swabbing and quantification process at the moment of sample collection (before chilling) but in later steps were able to growth due to recovery when collecting the post-chill sample. Nonetheless, this increase it can also be attributed to re-contamination at the hot box or during fabrication due to the manipulation of the carcass or simply by the ineffectiveness of subsequent interventions.

Statistical process control charts have a very close relation with hypothesis testing. The null hypothesis is that a process is under control while the alternative hypothesis is that the process is not under control. A point outside the limits established by the UCL and LCL can be considered as evidence against the null hypothesis but at the same time, it is also possible that this point is just error generated by random chance. The average run length (ARL) can describe the frequency with which theses errors occur. In a process that is under control, the probability that a point is outside the control limits (±3σ) is equal to 0.0027 (0.00135 + 0.00135); therefore, the ARL will be 370.4 (1/0.0027), meaning that it can be expected to find one sample every 370 samples that will plot outside the control limits just by random chance. In our examples, few observations are outside the UCL–LCL range which may lead to the conclusion that some of the steps are under control, but only 12 observations were collected during eight days. Therefore, more samples collected over longer time periods should be included in the analysis to make inferences that the process is under control, but it will be expected that if the process is out of control, the ARL will be less than 370.4.

## 4. Discussion

The comprehensive enumeration of microbial indicators throughout different steps in beef processing facilities (bio-mapping) is a novel approach for processors to have a general overview of how well their process performs in terms of achieving and maintaining low microbial loads. Bio-mapping studies allow processors to visualize upon data analysis the overall picture of which steps in the process are the most important for ongoing monitoring due to their effectiveness, and which ones can be removed or modified as their effect does not contribute to the overall reduction of microbial counts. Besides, microbial baselines developed by bio-mapping allows processors to make decisions based on ongoing comparisons with historic data of the process when process modifications are considered for implementation, including new processing schemes, new antimicrobials, or adjustments in speed lines, among others.

In this study, a clear trend for all indicator microorganisms for most of the different carcass regions sampled was observed with microbial reductions from pre-evisceration until final interventions, followed by an increment during chilling and fabrication. Enumeration of aerobic plate counts, Enterobacteriaceae, and *Escherichia coli* yielded reductions during the slaughter process in ranges of 1.7 to 2.44 Log CFU/cm^2^, 1.04 to 1.82 Log CFU/sample, and 0.41 to 1.43 Log CFU/sample, respectively. The results support the importance of using multiple-sequential interventions in beef packaging plants as a way to improve the microbiological quality of beef carcasses [18,24,25,26]. Multiple interventions that have been evaluated to control microbial levels on beef carcass, such as hot and cold-water washes, organic acid interventions, steam pasteurization, and knife trimming. Moreover, there are multiple studies that demonstrate their individual contributions at different steps during the slaughtering process, but also plenty experiments that report greater reductions when decontamination treatments are used in combination rather than individually [27]. The use of water washes followed by organic acids rinses have been proved to exert greater reductions in carcasses inoculated *Escherichia coli* O157:H7 and *Salmonella* typhimurium rather than when used individually [28]. The combination of organic acid interventions or ozone treatment with hot water washes demonstrated reductions of around 3.26 Log CFU/cm^2^ on beef trim in a commercial plant environment [9]. Furthermore, the combination of steam pasteurization and hot water wash proved to reduce *Escherichia coli* by 0.6 Log CFU/cm^2^ more than using the steam pasteurization or the water wash by itself [29]. Since the implementation of the zero tolerance requirements and the microbiological performance guideline, extensive research about the efficacy of interventions for beef processing facilities and strategies that focus on the concept of multi-hurdle technology always come up as the best solution to maintain microbial loads at safe levels [27,30,31].

Bio-mapping can also facilitate processors in understanding the performance of the process and can help the food safety team make data-based decisions on quality and safety aspects of beef meat. For all indicator microorganisms, swabs collected in the shank at pre-evisceration resulted in the higher counts; conversely, after evisceration, samples of the midline region presented the highest counts. The lowest counts during the process were observed after final interventions during slaughtering. The highest *Escherichia coli* counts were found in the brisket region of cold carcass, and for all indicator microorganisms, the highest counts were detected in the inside round after fabrication. This information provides a foundation to make decisions in support of food safety management programs, such as which carcass regions should be considered for sampling to analyze worst case scenarios, re-evaluation of interventions in the process, identification of key steps in which minimum changes may lead to dramatic non-compliance microbial standards in final product, or simply the understanding of increases in microbial levels due to the nature of the process in specific regions of the carcass. Furthermore, if there is an established sampling program and adequate analysis of the microbial data, this information can result in the implementation of targeted strategies such as antimicrobial interventions in specific areas of the carcass or different antimicrobial concentrations according to the region fabricated, that are based on risk and the likelihood of presence of high or low levels of microbial counts [32].

In addition to bio-mapping studies, processors have the opportunity to use the microbial counts data to statistical process control parameters, as a tool for microbial quality assurance in beef processing plants. The statistical process control parameters created for this study are a good initial estimation for the plant as few observations were observed outside the two boundaries parameters established in this study. However, it is recommended that a sound sampling plan is established to include more consecutive days (around 15 to 20) for robust decision making [22]. Additionally, it is also important to understand that microbial control limits have different interpretations than normal control limits as observations below the lower control limit (LCL) are not really observations out of control as lower counts than the lower limit represent better microbial performance. Indeed, these observations can be used as examples to track back and evaluate the good practices during that day that were responsible for such performance that can be later incorporated to the normal process. On the other hand, the upper control limit (UCL) can be used to explore occasions when the process is getting out of control and will provide support for considering corrective actions implemented in a timely manner to avoid great shifts in the process. In order to detect non-random conditions on control charts, there are decision rules that must be followed known as the “western electric rules” [22].

For small-sample process control charts, the first rule establishes that any observation falling above the +3σ limit should be considered as a point out of control [22,23]. Cold carcasses levels for all indicator microorganisms evaluated in this study will fall under this rule. The possible reason for this outcome is that in fact the process is out of control or that the number of samples taken for this experiment did not predict accurately the control parameters that measure the natural variance that exist in the process. Applying the first rule, 1 out of 7 sampling points were outside control parameters for APC, 3 out of 7 for EB, and 1 out 7 for EC. Six more rules exist that can be used to detect out of control situations that are related with the number of consecutive points falling in specific zones of statistical control charts (+1σ and +2σ boundaries). It is also important to highlight those results presented in this experiment are a representation of the process itself for this particular facility and should not be extrapolated to other companies with similar processes, as microbial performance is highly intrinsic to the different components, equipment, expertise during the slaughtering and processing of beef carcass, thus making every single process unique. The principal aim for the creation of statistical control parameters for this experiment was to provide potential users with a standardized protocol for handling microbial data, and manage it in a way that it can lead food safety personnel to make decisions that can improve the food safety system based on data that when combination with experience and strategic planning can result in beef carcasses with lower microbial counts, including pathogen loads that will contribute to the reduction of foodborne illnesses attributed to meat products.

## 5. Conclusions

Bio-mapping microbial indicators in this processing facility shows consistent reductions for all microorganisms evaluated up to the chilling location. Higher microbial loads were observed in all samples at pre- and post-evisceration with a continuous decrease as the process moved forward, until final interventions are applied. Then, a slight increase in counts occurred during the chilling process followed by a larger final increase after fabrication. The use of multi-hurdle interventions in the process can help with reductions in all indicator bacteria, which in turn can be translated in potentially lower levels of pathogenic bacteria. The variety of microbial loads obtained on different carcass regions throughout the process provides data to back up decisions on how to conduct microbial sampling based on worst case scenarios generated with data of their own process. The microbiological profile obtained in this facility not only provided a microbial baseline for supporting food safety continuous improvements to the plant, but also generated a comparative guideline for other beef processing facilities to evaluate the microbial control performance of their individual processes. The development of statistical process control parameters as a tool for food safety management to enhance the microbial performance on slaughtering and processing facilities resulted in basic reference values to the facility investigated to support their process improvements. However, more research and understanding of the process needs to be elucidated to interpret charts in applicable ways to evaluate the performance of food safety systems.

## Figures and Tables

**Table 1:** Summary table of indicator counts for all carcass regions at each sampling location throughout the beef processing line (n = 24 carcass region/sampling location).

Carcass Region	Sampling Location	Microorganism		
		Aerobic Plate Counts (Log CFU/cm^2^ ± SE ^1^)	Enterobacteriaceae Counts (Log CFU/Sample ^2^ ± SE)	*Escherichia coli* Counts (Log CFU/Sample ± SE)
Shank	Pre-Evisceration	3.93 ± 0.24 ^a^	3.47 ± 0.20 ^a^	2.83 ± 0.25 ^a^
	Post-Evisceration	2.58 ± 0.07 ^b^	2.55 ± 0.10 ^b^	1.78 ± 0.09 ^b^
	Final Rail	1.55 ± 0.13 ^c^	1.98 ± 0.16 ^c^	1.67 ± 0.11 ^b,c^
	After Interventions	1.49 ± 0.19 ^c^	1.65 ± 0.07 ^c^	1.40 ± 0.01 ^d^
	After Chilling	2.66 ± 0.17 ^b^	2.69 ± 0.23 ^b^	1.49 ± 0.06 ^c,d^
	Cold Carcass	2.77 ± 0.19 ^b^	3.25 ± 0.27 ^a,b^	1.65 ± 0.14 ^c,d^
	After Fabrication	1.60 ± 0.14 ^c^	2.67 ± 0.09 ^b^	1.58 ± 0.08 ^c,d^
	*p*-value	<0.001	<0.001	<0.001
Inside Round	Pre-Evisceration	3.49 ± 0.19 ^a^	2.93 ± 0.13 ^b^	2.72 ± 0.20 ^a^
	Post-Evisceration	3.19 ± 0.15 ^a^	2.91 ± 0.12 ^b^	2.58 ± 0.18 ^a^
	Final Rail	2.06 ± 0.14 ^b^	1.86 ± 0.12 ^c^	1.94 ± 0.12 ^b^
	After Interventions	1.24 ± 0.17 ^c^	1.47 ± 0.05 ^d^	1.45 ± 0.04 ^c^
	After Chilling	1.63 ± 0.25 ^b,c^	2.48 ± 0.27 ^c^	2.02 ± 0.23 ^b^
	Cold Carcass	1.81 ± 0.28 ^b,c^	2.42 ± 0.29 ^c^	2.23 ± 0.27 ^b^
	After Fabrication	3.26 ± 0.15 ^a^	4.00 ± 0.21 ^a^	3.22 ± 0.24 ^a^
	*p*-value	<0.001	<0.001	<0.001
Midline	Pre-Evisceration	2.94 ± 0.19 ^b^	2.57 ± 0.06 ^b^	1.87 ± 0.12 ^b^
	Post-Evisceration	3.79 ± 0.24 ^a^	3.81 ± 0.28 ^a^	2.79 ± 0.19 ^a^
	Final Rail	2.93 ± 0.23 ^b^	2.77 ± 0.21 ^b^	2.28 ± 0.20 ^b^
	After Interventions	1.66 ± 0.23 ^c^	1.53 ± 0.05 ^c^	1.46 ± 0.03 ^c^
	After Chilling	1.09 ± 0.12 ^c^	1.61 ± 0.13 ^c^	1.47 ± 0.06 ^c^
	Cold Carcass	1.30 ± 0.19 ^c^	1.79 ± 0.15 ^c^	1.53 ± 0.09 ^c^
	After Fabrication	2.72 ± 0.18 ^b^	3.55 ± 0.21 ^a^	2.22 ± 0.15 ^b^
	*p*-value	<0.001	<0.001	<0.001
Brisket	Pre-Evisceration	3.38 ± 0.18 ^a^	2.88 ± 0.12 ^b,c^	2.47 ± 0.16 ^a^
	Post-Evisceration	3.23 ± 0.20 ^a^	3.32 ± 0.21 ^a,b^	2.54 ± 0.21 ^a^
	Final Rail	2.39 ± 0.18 ^b,c^	2.41 ± 0.15 ^c,d^	1.92 ± 0.14 ^b^
	After Interventions	1.44 ± 0.12 ^d^	1.79 ± 0.12 ^e^	1.41 ± 0.01 ^c^
	After Chilling	2.34 ± 0.19 ^b,c^	2.54 ± 0.24 ^c,d^	1.58 ± 0.14 ^c^
	Cold Carcass	2.23 ± 0.24 ^c^	3.06 ± 0.27 ^b,c^	1.73 ± 0.18 ^c^
	After Fabrication	2.82 ± 0.16 ^a,b^	3.96 ± 0.20 ^a^	2.22 ± 0.15 ^a,b^
	*p*-value	<0.001	<0.001	<0.001

^1^ Standard error of the mean. ^2^ Log CFU/sample is equivalent to Log CFU/100 cm^2^ (detection limit = 0.01 CFU/cm^2^ or 25 CFU/sample). ^(a–d)^ For each carcass region and microorganism, values with different letters are significantly different according to ANOVA analysis followed by pairwise comparison *t*-test at *p* < 0.05 for Aerobic plate counts and Kruskal–Wallis analysis followed by pairwise comparison Wilcoxon’s test at *p* < 0.05 for Enterobacteriaceae and *Escherichia coli* counts.

**Table 2:** Statistical Process Control Parameters for Aerobic Plate Counts (Log CFU/cm^2^) at different sampling locations on the beef processing line. (n = 12 swabs/location collected during 8 different sampling days).

Sampling Location	Statistical Process Control Parameters (Log CFU/cm^2^)						
	$\overset{=}{X}$ − 3σ ^1^	$\overset{=}{X}$ − 2σ	$\overset{=}{X}$ − σ	$\overset{=}{X}$	$\overset{=}{X}$ + σ	$\overset{=}{X}$ + 2σ	$\overset{=}{X}$ + 3σ ^2^
Pre-Evisceration	2.54	2.84	3.18	3.44	3.74	4.04	4.34
Post-Evisceration	2.37	2.65	2.92	3.20	3.48	3.75	4.03
Final Rail	1.39	1.68	1.96	2.24	2.52	2.81	3.09
After Interventions	0.69	0.95	1.20	1.46	1.72	1.97	2.23
After Chilling	0.98	1.30	1.62	1.93	2.25	2.56	2.88
Cold Carcass	0.96	1.32	1.67	2.03	2.38	2.74	3.09
After Fabrication	1.75	2.04	2.32	2.61	2.90	3.19	3.47

$\overset{=}{X}$ = expected average value. σ = average standard deviation of the mean. ^1^ Upper limit (UCL). ^2^ Lower limit (LCL).

## Data Availability

Data are available on request from the corresponding author. The data are not publicly available due to privacy from the beef processing partner that allowed the project to be conducted within their beef processing environment.
